# Perceptions of pregnant women of reasons for late initiation of antenatal care: a qualitative interview study

**DOI:** 10.1186/s12884-020-2746-0

**Published:** 2020-02-03

**Authors:** Denis Warri, Asha George

**Affiliations:** 1 Cameroon Baptist Convention Health Services, P. O. Box 1, Bamenda, Cameroon; 2 School of Public Health, University of the Western Cape, Cape Town, South Africa

**Keywords:** Antenatal care, Maternal health, Pregnant women, Midwives, Care-seeking, Health education

## Abstract

**Background:**

Antenatal care serves as a key entry point for a pregnant woman to receive a broad range of services and should be initiated at the onset of pregnancy. The aim of the study was to understand the reasons for the late initiation of antenatal care by pregnant women in Nkwen Baptist Health Centre, Bamenda, Cameroon.

**Methods:**

The study applied purposive sampling to recruit eighteen pregnant women and three key informants for data collection through individual interviews. Pregnant women who initiated antenatal care after the first trimester were recruited during antenatal care clinics and were interviewed in a room at the Antenatal Care Unit. Key informants were midwives working at the Antenatal Care Unit. Participation in the study was voluntary. The purpose of the study was explained to participants, and they signed a consent form if they were willing to participate in the research. Data were audio-recorded and analyzed using thematic coding analysis.

**Results:**

Pregnant women placed a low value on early antenatal care because they perceived pregnancy to be a normal health condition or to not be a serious issue that required seeking health care. Furthermore, previous positive pregnancy outcomes for which women did not access care made them less motivated to initiate antenatal care early. Participants perceived the booking system to be user-unfriendly and complained of overcrowded conditions, long waiting times and some rude service providers. The cost of services and distance to health facilities that required travel via uncomfortable transport on poor road networks were identified as perceived barriers. The absence of effective community health programmes, perceived lack of support from parents and spouses, fear of bewitchment and stigma due to cultural beliefs about the early initiation of antenatal care were also identified as variables influencing late initiation.

**Conclusion:**

Pregnant women lack information on the purpose of early antenatal care. Health facility barriers as well as socio-cultural beliefs have significant influences on the timing of antenatal care initiation. The government of Cameroon should strengthen the health system and implement activities to engage communities in improving care seeking for antenatal care and thereby improving maternal health status of women.

## Background

In 2015, approximately 303,000 women died from pregnancy-related causes globally, with 99% of all maternal deaths occurring in low and middle-income countries [1]. Within low and middle-income countries, the highest maternal mortality rates are found in sub-Saharan Africa [2]. Within sub-Saharan Africa, the West African region has the highest maternal mortality in Africa, accounting for approximately 20% of global maternal deaths [3]. In West Africa, Cameroon has one of the highest maternal mortality rates, with 596 deaths / 100,000 live births [4]. Additionally, in Cameroon, approximately 80,000 women and girls suffer from injuries or disabilities caused by complications during pregnancy and childbirth each year [5]. The major causes of maternal mortality in Cameroon are haemorrhage, malaria, complications from unsafe abortion, hypertension, anaemia and pneumonia [6]. The skewed nature of maternal deaths demonstrates that the majority of maternal deaths could be prevented through timely interventions by skilled healthcare providers, whether during the antenatal, delivery or postpartum period [7, 8]. Antenatal care serves as a key entry point for a broad range of services that enable the detection and management of risky conditions associated with pregnancy and childbirth [8].

While research has demonstrated the benefits of antenatal care through the improved health of mothers and babies, the exact components of antenatal care and what to do at what time have been matters of debate [9]. Initially, the high-risk approach aimed to classify pregnant women as low risk and high risk based on predetermined criteria, which involved many visits [10]. This approach was difficult to implement effectively since many pregnant women have at least one risk factor, and not all women developed complications. At the same time, some low-risk women develop complications, particularly during childbirth [9]. After a 2001 systematic review, the World Health Organization (WHO) moved away from the high-risk antenatal care model developed largely for high-income countries [10]. The revised model was based on reduced but goal-oriented clinic visits (focused antenatal care), which consisted of at least four visits to a health facility during pregnancy [10]. More recently, evidence has shown that the focused antenatal care model is probably associated with more perinatal deaths than models that call for at least eight antenatal care visits [11].

Furthermore, evidence suggests that more antenatal care visits, irrespective of the resource setting, are probably associated with greater maternal satisfaction than fewer antenatal care visits [11]. Currently, the 2016 WHO antenatal care model states that antenatal care models with a minimum of eight contacts are recommended to reduce perinatal mortality and improve women’s experiences of care [11]. Research indicates that in low-income countries, particularly sub-Saharan African countries, pregnant women often do not receive the recommended antenatal care services [2]. To ensure that potential complications are identified in early pregnancy and managed effectively, the WHO recommends that women should initiate antenatal care early and have at least eight contacts with healthcare professionals during pregnancy [11]. In West Africa, many pregnant women, especially adolescent women, tend to start antenatal care late, resulting in them not benefiting from preventive and curative services. In a retrospective study on gestational age at antenatal booking and delivery outcomes in Nigeria, the results reported a prevalence of late booking of 86% [12]. This result is in line with a cross-sectional study in southern Benin. In this study in southern Benin, the primary target was pregnant women attending an antenatal care visit, regardless of the length of pregnancy. The results showed that among 301 pregnant women, only 24.6% utilized antenatal care services during the first trimester of pregnancy [13]. Another cross-sectional study in The Gambia showed similar results of high rates of late initiation of antenatal care. The study involved 457 women attending six urban and six rural antenatal clinics. The results indicated that only 8.1% of the women attended antenatal care visits within the first trimester of pregnancy, while 62.8 and 29.1% attended their first antenatal care visits in the second and third trimesters respectively [14]. Similar to other West African studies, research has shown that most pregnant women in Cameroon initiate antenatal care late [15, 16]. In a cross-sectional study in the Muea Health Area in the Southwest Region of Cameroon, which is made up of rural/semi-urban settlements, findings showed that only 27.2% of women had their first antenatal care visits in the first trimester [15]. Most of the women (69.1%) had their first visits in the second trimester, and 3.7% had their first antenatal care visits in the third trimester [15]. The research found that rural residence was associated with lower antenatal care attendance. Semi- urban women were more likely than rural women to initiate antenatal care early or attend four times or more. Financial constraints were the most significant barrier to the early initiation of antenatal care [15]. This was because in Cameroon, payment for services is out of pocket both in private and public health facilities with no exemption schemes. Community health insurance schemes are weak and not effectively utilized by the population. Similarly, the results of a cross-sectional study in a suburban hospital in Buea in the Southwest Region of Cameroon revealed that while 60.5% of women attended at least four antenatal care visits before delivery, only 20.5% of women attended antenatal care during the first trimester of pregnancy [16]. In contrast to the study in the Muea Health Area which is a rural/suburban area, in the study in Buea in the suburban hospital, socio-demographic and obstetric factors were not found to be associated with attendance of antenatal care in the first trimester [16].

The present study explored the perceptions of pregnant women concerning the late initiation of antenatal care and how their experiences influenced their decisions on the timing of antenatal care initiation. This article presents the methods and findings of the study and discusses these findings in relation to previous research on the late initiation of antenatal care.

## Methods

### Study design

The study was an exploratory, qualitative study given that it aimed to gain a deeper understanding of the perceptions, opinions and experiences of pregnant women and midwives regarding factors influencing early antenatal care initiation during pregnancy. Qualitative research can develop concepts that enable the understanding of social phenomena in a particular setting with emphasis on the meaning, experiences and views of participants [17]. Hence, the approach enabled the researchers to collect data through in-depth interviews with an interview guide using questions that were broad and open-ended to enable detailed exploration based on the responses provided. The approach also enabled the primary researcher to explore the reasons and opinions behind participants’ responses through asking probing “why”, “how” and “what” questions to gain a deeper understanding of the reasons for the late initiation of antenatal care among pregnant women.

### Research setting

The study was conducted in Nkwen Baptist Health Center, a semi-urban health centre located in the Bamenda Health District in the Northwest Region of Cameroon. Nkwen Baptist Health Centre is a faith-based outpatient clinic belonging to the Cameroon Baptist Convention Health Services. The health centre has 144 staff members and an average monthly patient attendance of 12,128. The average monthly antenatal care clinic attendance of pregnant women is 358. The cost to initiate antenatal care is at least 13,000 fcfa ($26) but slightly less in public health facilities. This cost excludes other costs, such as for transportation to the health facility and feeding during clinics. Payment for services is out of pocket both in private and public health facilities with no exemption schemes. The Bamenda Health District is an urban and semi-urban area. With approximately 337,036 inhabitants, the district has 17 health areas and covers a total surface area of 560 km^2^. There is one main hospital (Bamenda Regional Hospital), which functions as a referral hospital for 17 public, 12 lay private and 5 mission health facilities. The Bamenda Health District is located in the Northwest Region of Cameroon. With Bamenda as its capital city, the Northwest Region is the third most populated region in Cameroon, with an estimated population of more than 1.8 million inhabitants [18].

### Sampling and recruitment

The study sample comprised eighteen pregnant women and three key informant midwives. The inclusion criteria were pregnant women who presented for their first antenatal care after twelve weeks of pregnancy. The exclusion criteria were pregnant women who were less than eighteen years of age, and pregnant women who could not express themselves in English. Participants were selected through purposive sampling. They were asked some key demographic questions, including the number of weeks of gestation, to determine their eligibility for interviews. Key informants included Midwives serving at the Antenatal Care Unit. The inclusion criteria were midwives who had been serving in the antenatal clinic for at least two years. These midwives were included on the basis that they had been working and interacting with pregnant women and could provide information on their perceptions and views regarding the timing of antenatal care initiation. Interviewing midwives in addition to pregnant women was a means of triangulating data sources to improve the credibility of the findings.

Pregnant women who initiated antenatal care after the first trimester were informed about the study by service providers at the Antenatal Care Unit during the provision of antenatal care services. Respondents were only informed of the study at the end of their visits at the Antenatal Care Unit to ensure that the study did not interfere with their access to care. They were informed that participation was voluntary and that if they wished to participate, they would be referred to the primary researcher for interviews in a room in the clinic. Those who agreed to participate were given a piece of paper by the service providers to indicate that they were informed of the study and were directed to the primary researcher for interviews.

### Data collection method

Data collection was conducted through in-depth interviews. Interviews were conducted face to face. This method provided a rich form of data, as the participant was visible to the interviewer who could pick up on nonverbal cues. Questions were asked from a predetermined interview guide. The guide had a short list of questions with probes to help direct the interview in a particular direction in a conversational manner. Probing was a vital tool to ensure the credibility or true value of the data, as it allowed for the clarification of interesting and relevant issues raised by the respondent. The interviews were audio-recorded. This allowed the interviewer to prepare the transcript for analysis, based on a verbatim account of the interview. With the data recording, the interviewer was able to review the recording multiple times as needed to catch elements that were missed. Written notes were also used to record information as a supplement to the audio- recorded data. Data analysis was conducted alongside data collection and was stopped once saturation was reached. Each interview took between thirty minutes to one hour and was assigned a code and a date to maintain confidentiality. At the end of each interview, the audio recordings were transcribed verbatim by the primary researcher and analyzed manually using thematic coding analysis (TCA). The primary researcher’s diary notes were collated and analyzed at the end of each day to ensure reflexivity.

### Data analysis

Data analysis was performed manually using TCA. TCA is a form of inductive analysis in which categories or codes are allowed to emerge from the data [19]. The five phases of TCA are as follows: familiarization, coding, identification of themes, reviewing and refining, integration and interpretation [20]. The primary researcher continuously reflected on the setting and context to help interpret the phenomena. The primary researcher also drew on existing research to inform the interpretation as well as strengthen and support the argument.

### Ethical considerations

Participation in the study was voluntary for pregnant women and midwives. Respondents were informed of the study by staff only at the end of their visit at the Antenatal Care Unit to ensure that the study did not interfere with their access to care. Each respondent was provided with a letter explaining the study, requesting their participation and assuring them of the confidentiality of the study. Their consent was sought, and a consent form was available for them to sign if they were willing to participate in the research. Participation in the research did not inhibit the respondents’ access to care. The anonymity of the participants was ensured by not asking questions that revealed the identities of the participants and not linking the results to individual participants. Pseudonyms were also used in the presentation of findings to ensure anonymity. It was anticipated that the research would cause no harm to the research participants. However, a professional Counsellor from Nkwen Baptist Health Centre was available in case any of the pregnant women required emotional support or counselling as a result of the research process. Ethical clearance was obtained from the Biomedical Research Ethics Committee of the University of the Western Cape (UWC) and from the Institutional Review Board (IRB) of the Cameroon Baptist Convention Health Services. Administrative clearance was also obtained from the Director of Health Services of the Cameroon Baptist Convention which authorized the researcher to have access to the research participants at Nkwen Baptist Health Centre.

## Results

Eighteen pregnant women and three key informant midwives were interviewed. The socio-demographic details of the participants are summarized in Table 1. Pregnant women and midwives had the same opinions on the reasons for the late initiation of antenatal care. The results of the interviews are summarized according to the following themes:

**Table 1:** Socio-demographic information of participants

Description	*N* = 21
Age range (in Years)	n (%)
18–22	5 (23.8)
23–27	8 (38.1)
28–32	1 (4.8)
33–37	4 (19.0)
38–42	2 (9.5)
43–47	1 (4.8)
Marital status	
Single	4 (19.0)
Married	17 (81.0)
Gravidity	
1	5 (23.8)
2	7 (33.4)
3	4 (19.0)
4	4 (19.0)
5	1 (4.8)
Parity	
0	5 (23.8)
1	8 (38.1)
2	4 (19.0)
3	2 (9.5)
4	1 (4.8)
5	1 (4.8)

### Perceived susceptibility/perceived severity

- Value of early antenatal care
- Pregnancy as a normal health condition
- Misconception of the ideal booking time
- Obstetric history

### Perceived barriers

- Accessibility of antenatal care services
- High cost of initiating antenatal care
- Distance to health facility

### Cues for action

- Community health education

### Self-efficacy

- Pregnancy disclosure
- Support from spouse
- Reaction of parents

The Participants’ and primary researcher’s reflections about the setting and context were incorporated into the findings to provide a richer description of the perceptions of reasons for the late initiation of antenatal care.

### Perceived susceptibility/perceived severity due to late antenatal care initiation

Perceptions of susceptibility and severity are perceptions that the early initiation of antenatal care is not necessary or that there are no serious health implications of being pregnant that require the early initiation of antenatal care. Themes that emerged under this category of perceptions were: the value of early antenatal care; pregnancy as a normal health condition; and the ideal booking time.

#### Value of early antenatal care

Some of the pregnant women had the perception that the main purpose of the early initiation of antenatal care was to know the state of the baby and since the baby was not fully formed in the first trimester, they perceived the early initiation of antenatal care to be a waste of time or money.

I could not come for ANC by one or two months [of being pregnant] because the foetus was not yet formed so that I can do echography and know how the baby was doing. It was so early, so being so early like that it would have just been waste of time. (P1, single, age range 18-22, parity 0)

You need to go for antenatal care when pregnancy is big so that they can check the baby well. It’s just that when I hear someone saying they are going for antenatal at two or three months [of pregnancy], I judge that it’s because they have money to waste. I cannot just waste money like that. (P7, married, age range 18-22, parity 2)

Some women recognized the importance of early antenatal care but lacked insights into its purpose for pregnant women and instead had a general understanding that pregnancy required antenatal care at some point.

Early antenatal is good …because I am pregnant, and it [the antenatal clinic] is a place where, when you are pregnant and preparing to deliver, you must appear. Had it been that I was not pregnant, I could not be here, so I believe I am in the right place. (P8, married, age range 18-22, parity 1)

#### Pregnancy as a normal health condition

Many of the pregnant women considered pregnancy to be a normal life event rather than a condition that requires the attention of health personnel. Some of the pregnant women said they would seek antenatal care only if they felt unwell.

For me I don’t really see it that necessary to come for antenatal care clinic that early at two or three months because first of all am not sick, am just normal, am fine and there’s nothing wrong with me. (P17, married, age range 38-42, parity 3)

There was no problem within the first three months, so if there was a problem, that’s when I would have rushed and come earlier. (P2, married, age range 23-27, parity 0)

Key informants also said most of the pregnant women who initiated antenatal care late did so as a curative rather than a preventive service.

They [pregnant women who initiate antenatal care late] feel antenatal care is a curative issue, meanwhile that’s not the case. Antenatal care is preventive…this causes them to wait until they have a health problem before they come for antenatal care. (P19, married, age range 43-47, parity 5)

#### Misconception of the ideal booking time

Some women said that because the purpose of initiating antenatal care was to diagnose any problems that the baby may be having, the ideal booking time is after the first trimester when the baby is properly formed.

I know that it is normal [the ideal time] to come for antenatal clinic as from four or five months… at that time you can be able to know better how the baby is faring. (P13, married, age range 23-27, parity 2)

Many women did not have correct information on the ideal booking time due to misinformation from family members, or inadequate health education during clinics.

As I was growing up, my mother used to teach me all those things, that when a woman is pregnant, she needs to go for clinic from 4 to 5 months of pregnancy so that the nurses can know if the baby is doing fine (P2, married, age range 23-27, parity 1)

[The nurses] in the health talk [education] when I came here last time when I was pregnant [at five months] did not tell us that a pregnant woman should start clinic when she is just one, two or three months pregnant. I have not heard this before, it is very new to me. (P12, married, age range 23-27, parity 1)

#### Obstetric history

Women with a positive obstetric history perceived pregnancy and safe delivery to be a normal experience and did not see the need to initiate antenatal care early.

[As for previous deliveries], I did not have complications, am always fine. I always come for antenatal care clinic later than this [five months] usually seven months when its almost time for me to give birth. I have always been delivering safely so I have no problem … I believe is just going to be the same because the previous ones I just delivered safely, and this is even the fourth pregnancy. (P17, married, age range 38-42, parity 3)

Key informant midwives said that a positive obstetric history caused some pregnant women to see antenatal care as a routine and preferred to book later*.*

As women deliver more some of them think they know much and will not want to come and book earlier. They think that antenatal care clinic is just a routine, they just think that since they have been going for antenatal care clinic for the previous pregnancies there’s no need booking early. (PI20, married, age range 33-37, parity 4)

Among the pregnant women, one had a negative obstetric history. Due to her blood group and that of her spouse, all their previous children had sickle cell disease and did not survive. This influenced her to delay initiating antenatal care because she was contemplating terminating the pregnancy.

I lost two children in the past due to our electrophoresis status [the incompatibility of her blood group with that of her spouse]. I aborted the third and this is the fourth pregnancy and I am not happy about it all…I decided to come now because I was still thinking whether to keep the pregnancy or not [terminate the pregnancy]. (P16, married, age range 23-27, parity 0)

### Perceived barriers to antenatal care

This category refers to barriers that prevent pregnant women from initiating antenatal care early. Themes that emerged related to these perceived barriers were the accessibility of antenatal care services and distance to health facilities.

#### Accessibility of antenatal care services

Some women said the booking system was user-unfriendly, with long waiting times, and that some of the staff were rude, making accessing the services difficult and stressful. This influenced their timing of booking antenatal care.

The problem is the place is too congested, the population is too much. When you come you need to stand on a very long line and aahh its really stressful…standing on the long lines every month from the first month [of pregnancy] and for nine months is something I can’t really do….. So I decided to come from five months to the last month so that at least I will not have to stress a lot. (P11, single, age range 18-22, parity 0)

I was not really pleased with the way the welcome was at the clinic, some of them are very rude, they don’t take time to explain things and end up just shouting at us and that’s even the most reason why some of us don’t like to come early for clinic because we don’t want to interact with them. (P18, married, age range 38-42, parity 3)

#### High cost of initiating antenatal care

Some of the pregnant women said it was expensive to initiate antenatal care. They had to delay initiating care because they needed to plan and raise money to pay for the services.

Let me say within the first two months, things were really difficult for us, so even if I was to start by then, I woudn’t have started. Because you know the town is shaking [from socio-political tensions] now so everything is difficult. Money is difficult to get ……there would have been no money to pay for tests and drugs within the first two months. (P5, married, age range 23-27, parity 1)

Some women said they could not afford to pay for antenatal care services and delayed initiating antenatal care to reduce the number of clinic visits, thereby reducing the total cost of antenatal care over the entire pregnancy.

You know there are financial challenges, there is a lot of hardship here and you have to pay for the cost of antenatal care … to start coming from the first month [of pregnancy] to the last month like that I don’t really have money because it is expensive to be coming from the first month to the last month, no, no, I cannot afford money to pay. (P17, married, age range 38-42, parity 3)

Midwives concurred that initiating antenatal care was expensive ($26) for many pregnant women and that lack of finances was one of the reasons why many of them booked late. This amount was too high for women within this community to afford to initiate antenatal care.

For first booking you spend at least 13,000 FCFA [$26] and they [pregnant women] always see early booking to be expensive to them…… we always at least attend to them and give them services according to the money they are able to have and tell them to go and look for money and come and finish their lab tests. (P21, single, age range 33-37, parity 1)

#### Distance to health facility

Some women said that the distance to the health facility was far and that transportation difficulties to reach the facility caused them to postpone initiating antenatal care early.

I do have difficulties of transport to come for clinic. You know the distance is far and I use bike [motor cycle], am always very dizzy, that makes it difficult [to initiate antenatal care early] (P11, single, age range 18-22, parity 0)

Even though we have tarred road but the only means of transport is [by] bike we don’t have taxi. It’s difficult with this pregnancy to climb on a bike, you are not comfortable, you are not sitting well so most at times you find yourself trekking for long to where you can see a taxi to come for the clinic …when you just think how you start trekking or climbing on a bike and start rolling down a long distance with all the wind it discourages you from going [to initiate] antenatal care early. (P18, married, age range 38-42, parity 3)

### Cues for action

Cues for action refer to triggers that can cause a pregnant woman to take necessary action to initiate antenatal care early. The absence of these cues can cause pregnant women to initiate antenatal care late. The theme that emerged under cues for action was community health education.

#### Community health education

The absence of effective community outreach programmes that could make women aware of the need to initiate antenatal care early caused some pregnant women to initiate late.

To say health workers come to the community to educate us on how to go about [early antenatal care initiation] when you are pregnant, I have not seen that… [As far as] seeing a doctor or a nurse coming around our quarter to help enlighten [educate] us on pregnancy and [early] antenatal care, I have never seen [that]. (P18, married, age range 38-42, parity 3)

These pregnant women are ignorant of things about pregnancy and [early] antenatal [care], where we can really educate women about them, they lack education…we lack a community forum [to have education] on early start of antenatal clinic… (P19, married, age range 43-47, parity 5)

### Self-efficacy

Self-efficacy refers to the confidence that enables a pregnant woman to be motivated to take action. It is influenced by socio-economic and demographic factors. In this study, some pregnant women did not believe that they were capable of making the decision to initiate antenatal care within the first trimester. Themes that emerged under self-efficacy were the cost of initiating antenatal care, pregnancy disclosure, support from spouse, and the reaction of parents.

#### Pregnancy disclosure

Some of the participants initiated antenatal care late because they wanted to delay making the pregnancy public, because of fear of perceived “enemies” who could harm their pregnancies.

I did not come before this time because I did not want people to know especially people who don’t wish me well, my enemies. (P2, married, age range 23-27, parity 1)

Other women said they delayed making their pregnancy public because they were shy or ashamed when the pregnancy was still early. It was noted that stigma associated with early pregnancy disclosure influenced both married and unmarried women in relation to the timing of antenatal care booking.

Pregnancy in our culture even though you are married it has some types of conceptions. At times I am shy and so I will not want my neighbours and people around to first of all know …. Culturally you feel shy…. even though [you are] married, it has a little aspect of shame related [to it]. You don’t feel comfortable you just feel a type [uncomfortable]. (P18, married, age range 38-42, parity 3)

One of the key informant midwives said that unmarried women especially young girls also hide the pregnancies within the first trimester due to the shame that information about their pregnancies will bring to their parents.

Most pregnant women at the beginning of pregnancy are always shy especially those who are not married, they shy away first of all because they don’t want their neighbours, or their immediate family members to know that they are pregnant so they hide the pregnancy seriously…some are ashamed for fear of stigma that their neighbours will laugh at their parents that though she was so holy she is not married but is pregnant. (P19, married, age range 43-47, parity 5)

Some of the women said community members considered early antenatal care to be a show of pride and mocked women who initiated antenatal care early.

So for us we believe that you only start going for clinic when the stomach is already very big [such] as such from six months. Because when you go for antenatal care at one or two months when the baby is still small, it is like you are boasting of something, proud which does not really speak well of you[in the community]. (P17, married, age range 38-42, parity 3)

#### Support from spouse

In some cases, lack of support from the spouse contributed to late initiation. Lack of trust made some husbands not believe their wives when their wives told them that they were pregnant. This made the husbands reluctant to provide money for the early initiation of antenatal care.

Whenever I tell the father of my children that I am pregnant, he usually takes it for a lie…each time I request for money to go for clinic he is not willing and will ask me to wait and he will give it [money] at his own time. (P7, married, age range 18-22, parity2)

Lack of knowledge on the ideal booking time by husbands also contributed to them providing less support to their wives to initiate antenatal care early.

I was not given money on time by my husband and when I said I was pregnant and needed to go for antenatal care early, he thought I was lying… It took many months before he gave me money….. He thought one needed to go for antenatal care at 6 months [of pregnancy]. (P14, married, age range 18-22, parity 0)

Marital misunderstanding was also identified as one of the reasons that caused many husbands not to support their wives to book early.

Sometimes he [my husband] is not understanding, what I will actually want from him he will not even give me. Like this food they are telling us to go and eat, I don’t know how I will explain to him because according to him he thinks that I just want to take his money and eat… he [will] just get angry and say, “Why are you struggling to go? You just want to waste my mone”. (P18, married, age range 38-42, parity 3)

#### Reaction of parents

Most of the unmarried women especially young girls said that the fear of negative reactions from their parents led to the late disclosure of pregnancy and hence contributed to the late initiation of antenatal care.

My parents were not going to welcome the pregnancy since I was just a student …so telling them when the pregnancy was still one or two months or so it would have been a taboo or something and I will surely be beaten…my parents are wild and they could do anything, so I was scared [and decided to hide the pregnancy from them]. (P11, single, age range 18-22, parity 0)

## Discussion

Our study identified the following four key themes that we used as a basis to explain the reasons for late initiation of antenatal care: perceived susceptibility/perceived severity due to late antenatal care initiation; perceived health system barriers to early antenatal care; cues for action; and self-efficacy. Explanatory aspects of the findings are elaborated and placed in the context of the broader literature.

### Perceived susceptibility/perceived severity due to late antenatal care initiation

A major finding of the study was the lack of knowledge on the purpose of early antenatal care and therefore the right time to initiate antenatal care. This lack of understanding is influenced by a perception that antenatal care is primarily provided to detect or treat diseases. This explains why many participants said they did not have any problems in early pregnancy that required the intervention of health personnel. Some respondents assumed that there were no benefits in booking in the first three months. There is a perception that women can successfully go through the first trimester of pregnancy without antenatal care. Pregnant women view health issues as normal health conditions or not serious enough to require that they seek healthcare. These are the arguments used by those advocating for goal-oriented antenatal care visits. Hence, antenatal care is perceived as a curative rather than a preventive intervention. This is in line with a study by Ndidi and Oseremen in which they reported that most women booked antenatal care late because of the belief that there are no advantages to booking antenatal care in the first three months of pregnancy [21]. Some of the women were aware of the importance of early antenatal care but lacked insight into its comprehensive purpose. The value of the early initiation of antenatal care was not well described, and descriptions of antenatal care most often focused on curative care or preparation for delivery, as was found in a study in rural South Africa [22].

In this study, some participants believed that there was no ideal booking time for antenatal care. This is similar to a study in southern Nigeria in which the majority of pregnant women claimed that it is safe to book antenatal care at any time during pregnancy [23]. There were diverse reasons for the lack of information on the ideal booking time. Some participants responded that they had never been informed of the ideal booking time by service providers during previous antenatal clinics. Health education programmes during antenatal care clinics failed to address the issue of the ideal booking time, and multi gravidas who booked late in previous pregnancies were likely to continue with the same practice during subsequent pregnancies. In a study in Buea Health District in Cameroon, few and ineffective health education sessions held by service providers during antenatal care clinics were highlighted to be related to the poor utilization of antenatal care services by pregnant women [15]. It has been found that past experience with antenatal care services is not a predictor of the timely booking of antenatal care [24].

In this study, some participants responded that they grew up observing their mothers initiating antenatal care later in pregnancy. Other participants said they were advised by their mothers or spouses to initiate antenatal care after the first trimester. The study reveals the important role that parents or spouses play in deciding the time of booking antenatal care. There is a need to develop health education programmes that empower parents and spouses to improve their knowledge of the importance of early antenatal care services. In a study in southwestern Nigeria, incorrect advice on the best time to start antenatal care from relatives or partners was highlighted as one of the reasons why women in their first pregnancies started antenatal care late [25].

Multigravida participants said they used previous positive pregnancy outcomes as experience in handling subsequent pregnancies. Previous positive pregnancy experiences made pregnant women develop confidence and thus were less motivated to initiate antenatal care early. This is in line with a study that found that multiparous women were usually confident, believing that having delivered many times previously, they were well versed in pregnancy and delivery and did not need to book antenatal care early [23]. On the other hand, previous negative pregnancy outcomes influenced some of the participants to delay initiating antenatal care because initially, they planned to terminate the pregnancy. This finding is consistent with those of a study that found that some women postponed initiating antenatal care until they were free from a perceived obligation to terminate the pregnancy. This may occur with unplanned pregnancies among women with difficult obstetric histories [26]..

### Perceived health system barriers to early antenatal care

Some respondents perceived the booking system to be user-unfriendly. They complained of overcrowded conditions; many movements between the consultation room, laboratory, ultrasound room, and pharmacy, which were far from each other; long waiting times; and some rude clinic staff. These experiences undermined the quality of antenatal care offered to pregnant women. Women who perceived poor quality services preferred to delay the initiation of antenatal care to avoid going through the experience at the early stage of pregnancy.

Dissatisfaction with care in health facilities, including long waiting times and, rude and unfriendly attitudes of healthcare providers has been found to be related to late booking among pregnant women [27].

In this study, some participants expressed their inability to afford the cost of initiating antenatal care and the necessity to delay booking until they raised the required amount. While some women said the cost of initiating antenatal care ($26) was expensive, others said the negative economic effects of the socio-political tensions in the region aggravated their financial hardship, limiting their ability to pay for the cost of booking antenatal care early. Booking for antenatal care requires payment for a number of laboratory tests and drugs. In addition, pregnant women have to pay for transport to the health facility. Most of the women in this community are poor. Payment for services is out of pocket, and there are no exemption schemes. This system renders many women unable to afford for health services. In a low-resource setting such as Cameroon, financial constraints and distance to the health facility play a major role in determining the timing of initiation of antenatal care. Distance limits women’s ability and willingness to seek health care, as the road network is poor, and the common means of transport is by motorcycles. These reasons are similar to those observed in a study conducted in Ethiopia, where financial constraints were amongst the most common reasons for late antenatal care booking [28]. Tolefac et al. also found that in Cameroon, distance to the nearest health facility and transport cost were strong barriers to the early initiation of antenatal care among pregnant women [29]. Uncomfortable transport and poor road conditions have also been found to be barriers to the utilization of antenatal care by pregnant women [30].

### Cues for action

This study found that there was no effective community outreach to serve as cue for action for pregnant women to initiate antenatal care early. The absence of a community health education programme contributed to the lack of knowledge on the ideal booking time, which led to late initiation of antenatal care by pregnant women. If women are to be encouraged to seek antenatal care early, the purpose and value of the early initiation of antenatal care will need to be communicated across the communities in which they live. Other studies have found that public health strategies within communities are necessary to raise awareness and promote early antenatal care services among pregnant women [31].

### Self-efficacy

In this study, fear of disclosing pregnancy due to community pressures and beliefs was associated with the late initiation of antenatal care. Some participants delayed the initiation of antenatal care out of shame, while others were afraid of being mocked by community members for initiating antenatal care too early. Fear of bewitchment was also mentioned as a reason for booking antenatal care late by some women. Fear of perceived “enemies” who could harm a woman’s pregnancy has been found to contribute to the late initiation of antenatal care [21]. These findings may also support previous findings that social norms such as seeking advice from village elders before disclosing pregnancy are still dominant in the decision-making process regarding the timing of antenatal care initiation [32]. Some participants indicated that unplanned pregnancies, especially among young single women, were in most cases associated with late disclosure to parents due to fear of potentially negative reactions. A perceived lack of parental support translated into the late initiation of antenatal care. Social support has been shown to facilitate early antenatal care attendance [33]. Lack of support from spouses through their refusal to provide money required to cover the cost of antenatal services or their discouragement of the early initiation of antenatal care was highlighted by some of the participants as reasons for the delayed initiation of antenatal care. These women had to wait for their spouses to decide for them about when to start attending the clinic. Their spouses either did not provide the cash to cover the cost of antenatal care or were ignorant of the importance of early antenatal care. In Cameroon, husbands play a key role in decision making for women, hence the need to involve men in health education programmes that aim to promote the effective utilization of antenatal care services. Having a spouse who is not supportive was highlighted as being associated with initiating antenatal care late for both adolescent and adult pregnant women in southeastern Tanzania [34].

## Conclusion

The study explored the perceptions of pregnant women of reasons for the late initiation of antenatal care. In Cameroon only approximately 20.5% of pregnant women initiate antenatal care within the first trimester of pregnancy. The study showed that pregnant women and midwives have the same opinions of the reasons for the late initiation of antenatal care:

- Pregnant women place a low value on early antenatal care due to a lack of knowledge of its importance. They perceive pregnancy to be a normal health condition or to not be a serious issue that requires seeking health care.
- Pregnant women lack information on the ideal booking time due to the ineffectiveness of health education programmes during antenatal care clinics. Misinformation from family members and spouses is also a reason for the lack of information about the ideal booking time for antenatal care among pregnant women.
- Some participants perceive the booking system to be user-unfriendly and complain of overcrowded conditions, long waiting times and rude clinic staff.
- Women who perceive poor quality services prefer to delay initiating antenatal care to avoid going through the experience at the early stage of pregnancy.
- The high cost of initiating antenatal care as well as the distance to health facilities that require travel via uncomfortable transport and poor road networks are also identified as barriers to the early initiation of antenatal care.
- The absence of effective community health education programmes that could serve as triggers for early antenatal care contributed to a lack of knowledge of the ideal booking time. This causes some pregnant women to initiate antenatal care late.
- A perceived lack of support from parents for unmarried young women and a lack of support from spouses for married women lead to the late initiation of antenatal care. Fear of bewitchment and stigma due to cultural values and beliefs about the early initiation of antenatal care are also variables influencing late initiation.

These findings are the same with those found by other researchers more than twenty years ago. The major question for reflection is whether global development goals have made any difference in maternal health services and outcomes in countries such as Cameroon. The government of Cameroon should effectively implement activities that engage communities in improving care seeking for antenatal care and thereby improving the health status of women. Service providers should also implement strategies that will strengthen health facilities to provide quality services to pregnant women.

## Data Availability

Not applicable

## Abbreviations

ANC: Antenatal Care
HIV: Human Immunodeficiency Virus
IEC: Information Education and Communication
IRB: Institutional Review Board
P: Participant
PMTCT: Prevention of Mother to Child Transmission
TCA: Thematic Coding Analysis
UNICEF: United Nations Children’s Fund
USAID: United States Agency for International Development
UWC: University of the Western Cape
WHO: World Health Organization
